# A primary care approach to abdominal pain in adults

**DOI:** 10.4102/safp.v63i1.5280

**Published:** 2021-03-10

**Authors:** Indiran Govender, Selvandran Rangiah, Tombo Bongongo, Philemon Mahuma

**Affiliations:** 1 Department of Family Medicine, School of Health Sciences, University of Pretoria, Pretoria, South Africa; 2 Department of Family Medicine, School of Public Health, University of KwaZulu-Natal, Durban, South Africa; 3 Department of Family Medicine, School of Medicine, Sefako Makgatho Health Sciences University, Ga-Rankuwa, South Africa

**Keywords:** abdominal pain, primary care, pathophysiology, history guide, examination guide, diagnostic approach

## Abstract

Abdominal pain is a common presenting problem with multiple aetiologies that often pose diagnostic and therapeutic dilemmas for primary care practitioners. The vague symptomatology and difficult correlation to specific organ pathology obscures clinical findings leading to incorrect diagnoses. Although most presentations of abdominal pain are benign, a significant number of patients have life-threatening conditions that require a meticulous approach to management in order to prevent morbidity and mortality. The skill in assessing patients presenting with abdominal pain is fundamental for all primary care doctors. This review will discuss an approach to the assessment and diagnosis of abdominal pain in the primary care setting.

## Introduction

Abdominal pain is one of the most common complaints of patients admitted to emergency units, accounting for 5% – 10% of all presentations.^1,2,3^ Evaluation of the emergency department patient with acute abdominal pain may be difficult as several factors can obscure the clinical findings resulting in incorrect diagnoses and subsequent adverse outcomes.^4^ Primary care practitioners must therefore consider multiple diagnoses whilst giving priority to life-threatening conditions that require meticulous management to prevent morbidity and mortality. Skill in the assessment of a patient presenting with abdominal pain is essential for all primary care doctors.

The diagnostic challenge facing primary care physicians regarding patients with abdominal pain, considering the spectrum of symptoms, diagnoses and management, presents a potential risk of delaying treatment for acutely ill patients.^5^

This review seeks to provide an understanding of the pathophysiological basis and an approach to assessing the cause of abdominal pain in adults by primary care practitioners. Key aspects of history and physical examination will be discussed with the view to enhance appropriate and accurate assessments, management and early referral to higher levels of care.

## Pathophysiology

Abdominal pain may originate from within the peritoneal cavity, the retro peritoneum, the pelvis, the abdominal wall or even from outside the abdomen. The physiological basis for intra-abdominal pain is listed in Table 1.

**TABLE 1:** Pathophysiology of abdominal pain.

Process	Example of disorders
Inflammation	Appendicitis; cholecystitis; pancreatitis; diverticulitis.
Perforation	Perforated duodenal or gastric ulcer; biliary peritonitis.
Obstruction	Acute small or large bowel obstruction; biliary or ureteric colic.
Haemorrhage	Ruptured ectopic pregnancy; ruptured aneurysm or ovarian cyst; spleen.
Torsion (ischaemia)	Sigmoid volvulus; torsion of testes; ovarian cyst.

*Source:* Murtagh J. John Murtagh’s general practice. 4th ed. Sydney: McGraw-Hill; 2007.

Pain receptors in the abdomen are stimulated by mechanical and chemical stimuli. Stretch is the primary mechanical stimulus whilst visceral mucosal receptors respond to chemical stimuli.^7^ The precise events responsible for the perception of abdominal pain is not well understood but depend on the type of stimulus and the interpretation of visceral nociceptive inputs in the central nervous system.

Localisation of visceral pain is ill-defined because of the type and density of visceral afferent nerves. The pain is usually perceived in the midline because most abdominal organs are innervated by afferent nerves from both sides of the spinal cord.^7^ However, pain that is lateralised may arise from the ipsilateral kidney, ureter or ovary.^7^ A summary of the mechanisms of abdominal pain is presented in Table 2.

**TABLE 2:** Mechanisms of abdominal pain.

Mechanism	Cause	Innervation	Nature	Location
Visceral	Inflammation, ischaemia, neoplasia and distension of either the wall of a hollow viscus, or the capsule of a solid intra-abdominal organ.	Afferent nerves from either side of the spinal cord	Colicky, cramp-like dull and burning, often with associated autonomic symptoms of nausea, vomiting, pallor and sweating.	Poorly demarcated; usually midline via autonomic fibres in the wall or capsule. Regional localisation to foregut, midgut and hindgut structures
Parietal/somatic	Inflammation (bacterial or chemical) of the parietal peritoneum	Mediated by segmental nerves associated with specific dermatomes	Sharp aggravated by movement, coughing and breathing	Precise location to the structure of origin
Referred	Infection, infarction, embolism, irritation; shares common embryological origin	Peripheral nerves sharing a common central pathway	Dull, aching perceived near the surface of the body; skin hyperalgesia. Increased muscle tone	Localised to a site distant to organ that is the source of pain

*Source:* Murtagh J. John Murtagh’s general practice. 4th ed. Sydney: McGraw-Hill; 2007.

Abdominal wall pain is frequently mistaken for intra-abdominal visceral pain with consequence of unnecessary investigations, imaging and procedures.^8,9^ Hence an understanding and awareness should be part of a complete assessment.

Abdominal wall pain comprises a number of aetiologies of which nerve entrapment, hernias and procedural complications are common.^10^

Anterior cutaneous nerve entrapment syndrome is the most common and often passed-over type of abdominal wall pain.^11^ This condition typically presents with acute or chronic localised pain at the lateral edge of the rectus abdominis that worsens with positional changes or increased abdominal muscle tension.^12^ Abdominal wall pain should be suspected in patients with no symptoms or signs of visceral aetiology and a localised small tender spot. A positive Carnett test, in which tenderness stays the same or worsens when the patient tenses the abdominal muscles, suggests abdominal wall pain.^8^

## History

The cornerstone of an accurate diagnosis is a detailed history that includes a full description of the patient’s pain and associated symptoms. The medical, surgical and social history may provide valuable information to aid assessment.^4^

The PQRST mnemonic illustrated in Table 3 is a helpful reminder of a complete history.^4^

**TABLE 3:** Pain assessment history.

Pneumonic	Pain assessment
P3	Position, palliation and provoking factors
Q	Quality
R3	Region, radiation and referral
S	Severity
T	Temporal factors (time and mode of onset, progression and previous episodes.

*Source:* McNamara R, Dean AJ. Approach to acute abdominal pain. Emerg Med Clin N Am. 2011;29(2):159–173. https://doi.org/10.1016/j.emc.2011.01.013

The ‘PHRASED’ approach in gathering sufficient information is a useful guide to exploring the *Patient’s problem, History of the presenting problem, Relevant medical history, Allergies, Systems review, Essential family and social history* and the use of *Drugs.*^9^

In addition, Box 1 adapted from Murtagh’s general practice is a helpful reminder of specific questions to ask whilst gathering sufficient and detail history.

BOX 1 Key questions to ask in the history.

- What type of pain is it: Is it constant or does it come and go?
- How severe would you rate it from 1 to 10?
- Have you ever had previous attacks of similar pain?
- What else do you notice when you have the pain?
- Do you know of anything that will bring on the pain? Or relieve it?
- What effect does milk, food or antacids have on the pain?
- Have you noticed any sweats or chills or burning of urine?
- Are your bowels behaving normally? Have you been constipated or had diarrhoea or blood in your motions?
- Have you noticed anything different about your urine?
- What medications do you take?
- How much aspirin do you take?
- Are you smoking heavily or taking heroin or cocaine?
- How much alcohol do you drink?
- How much milk do you drink?
- Have you travelled recently?
- What is happening with your periods? Is it mid-cycle or are your periods overdue?
- Does anyone in your family have bouts of abdominal pain?
- Do you have a hernia?
- What operations have you had for your abdomen?
- Have you had your appendix removed?

*Source*: Murtagh J. John Murtagh’s general practice. 4th ed. Sydney: McGraw-Hill; 2007.

It is important to consider the past medical history, as it will guide the possible cause of the abdominal pain. The important reminders are listed in Box 2.^6^

BOX 2 Past medical history reminders.

**Table:** 

Depression	Thyroid disorder	UTI
Diabetes	Spinal dysfunction	Herpes zoster
Drugs	Anaemia	Pleurisy

*Source:* Murtagh J. John Murtagh’s general practice. 4th ed. Sydney: McGraw-Hill. 2007.

UTI, urinary tract infection.

**FIGURE 1:**
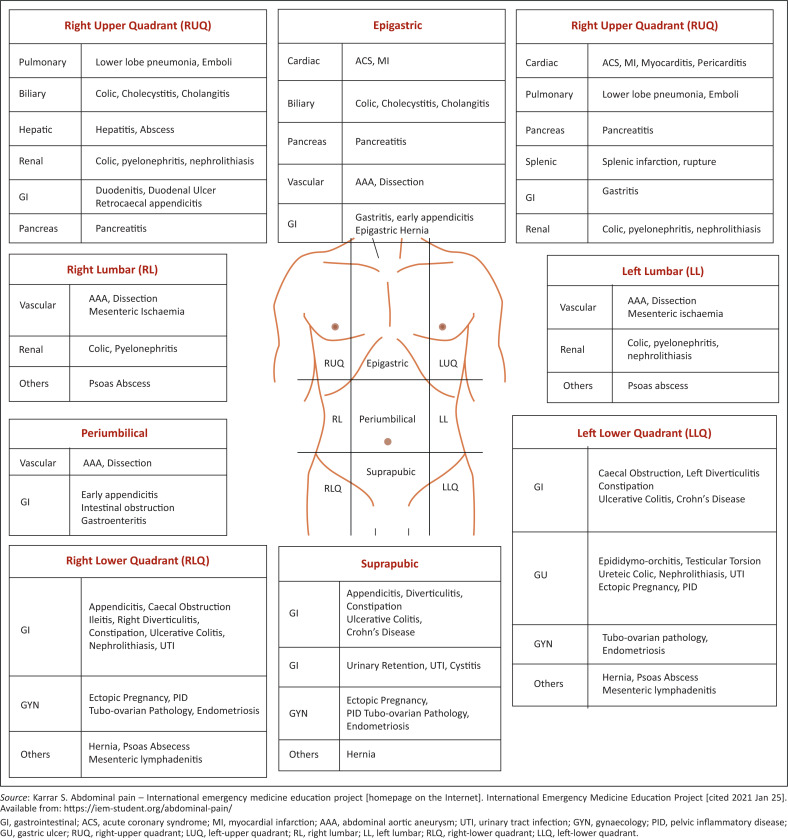
Anatomical localisation of pain.

Although the site of pain is important in identifying the source, visceral pain, referred pain or pain as a result of metabolic, toxic or psychological cause are not site dependent. Separating the abdomen into nine regions helps in describing the position of pain, tenderness, rigidity, tumours, et cetera. A summary of pathologies related to the anatomical regions of the abdomen is illustrated in Figure 1.

## Examination

A well-performed abdominal examination provides diagnostic clues regarding most gastrointestinal and genito-urinary pathologies decreasing the need for expensive radiological investigations.^14^

It is important that all healthcare workers are skilled in performing a correct abdominal examination, understand significance of findings and correlate these with the history in order to formulate a diagnostic strategy and management plan.^14^

This review does not provide a detailed discussion on abdominal examination.

The objectives of the abdominal examination includes assessment of the patient’s general condition, including a primary assessment, localisation of intra-abdominal pain and detection of extra-abdominal cause of pain.^8^

The patient’s general appearance and vital signs will guide to the differential diagnosis. Patients with peritonitis tend to lie still, whilst those with renal colic seem unable to stay still.^15^

Some often overlooked manoeuvres are useful in evaluating signs associated with the causes of abdominal pain. The Carnett’s sign – increased pain when a supine patient tenses the abdominal wall by lifting the head and shoulders off the examination couch is suggestive of abdominal wall pain.^16,17^ Others include Murphy’s sign for cholecystitis and the psoas sign for appendicitis.^18,19^

In diagnosing abdominal pain, rectal and pelvic examinations are mandatory. A rectal examination may indicate faecal impaction, a palpable mass, or occult blood in the stool whilst tenderness and fullness on the right side of the rectum suggest a retrocaecal appendix.^15,20^ A pelvic examination may reveal vaginal discharge suggestive of vaginitis. The presence of cervical motion tenderness and peritoneal signs increase the probability of ectopic pregnancy or other gynaecologic complications, such as salpingitis or a tubo-ovarian abscess.^15,21^

## Investigations

Imaging and laboratory studies have significant images in the evaluation of acute abdominal pain and all diagnostic tests have a false negative rate.^22^ The use of plain abdominal X-rays has not improved diagnostic accuracy, and instead has resulted in unnecessary costs and increased radiation exposure.^23^

The choice of laboratory investigations is therefore driven by the clinical situation to confirm differential diagnoses deduced from a good history and examination. These may include a Full Blood Count, inflammatory markers like C-reactive protein (CRP) and erythrocyte sedimentation rate (ESR), liver function tests, amylase and lipase, blood glucose, urine analysis, pregnancy test and faecal blood and arterial blood gas.

The main indications for plain x-rays include:^12^

- Intestinal obstruction
- Perforated viscus
- Foreign body
- Renal/ureteric colic
- Chest pathology

Ultrasound is widely used in the assessment of abdominal pain as it is non-invasive and has no radiation risk.^24^ Although, the inappropriate use can delay diagnosis, there is evidence of the diagnostic and therapeutic impact of ultrasound in the setting of abdominal pain.^25^

Urgent ultrasound scan is useful in the following situations:^12^

- Suspicion of abdominal aortic aneurysm
- Suspicion of intra-abdominal abscess
- Suspicion of cholelithiasis with right upper quadrant pain
- Suspicion of urinary tract obstruction
- Lower abdominal pain in fertile women

The use of algorithms can serve as a useful guide to approaching abdominal pain depending on the anatomical site of origin of pain. Vaghef-Davari et al. have developed several algorithms for the management of abdominal pain.^2^

**FIGURE 2:**
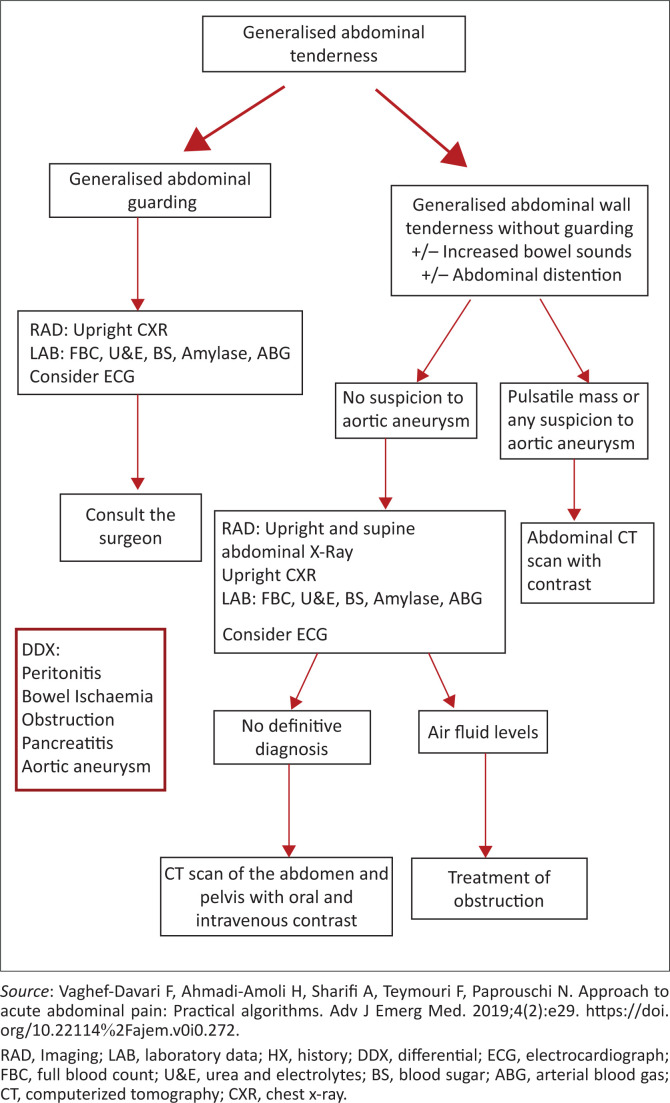
Generalised abdominal pain algorithm.

An illustration of the approach to generalised abdominal pain adapted from Vaghef-Davari et al. is shown in Figure 2.

## Management

The management of abdominal pain must be initiated simultaneously with assessment and investigations. Analgesia, fluid resuscitation, anti-emetics, nasogastric suction and antibiotics form the basis of most management plans, depending on the clinical findings following the history, examination and investigations.

Early appropriate analgesia reduces suffering and often aids in better co-operation of the patient in providing a better history and responses to examination. Opioid analgesia is not a contraindication and the previous injunction that it may mask the correct diagnosis of abdominal pain is unfounded.

A rapid diagnosis and immediate treatment are required for patients who may have life-threatening conditions,^12^ which include patients with:

- Airway compromise with recurrent vomiting, or altered level of consciousness
- Requiring oxygen and/or ventilation
- Signs of circulatory failure.

## Definitive management

After secondary assessment and emergency treatment, some patients with abdominal pain will require referral to the next level of care.

Indications to higher level of care include:^12^

- Suspected generalised tenderness
- Suspected bowel obstruction
- Tenderness with uncontrolled vomiting
- Suspected pancreatitis
- Suspected aortic aneurysm
- Gastrointestinal bleeding
- Associated mass
- Severe pain with no confirmed cause
